# *In silico* identification of natural compounds against SARS-CoV-2 main protease from Chinese herbal medicines

**DOI:** 10.2144/fsoa-2023-0055

**Published:** 2023-06-14

**Authors:** Yi Kuang, Wenjing Shen, Xiaodong Ma, Ziwei Wang, Rui Xu, Qingqing Rao, Shengxiang Yang

**Affiliations:** 1 College of Chemical & Materials Engineering, Zhejiang A&F University, Lin'an, 311300, Zhejiang, China

**Keywords:** Chinese herbal medicine, Japonicone G, molecular docking, molecular dynamics simulation, SARS-CoV-2 Mpro

## Abstract

**Aims:**

To determine natural compounds with inhibitory effects toward SARS-CoV-2 Mpro from Chinese herbal medicines.

**Materials & methods:**

∼1200 natural compounds from 19 Chinese herbal medicines were collected. Computational methods including molecular docking, drug-likeness assessment, molecular dynamics simulation and molecular mechanics Poisson-Boltzmann surface area analysis were combined to obtain potent inhibitors against SARS-CoV-2 Mpro.

**Results:**

Top 20 compounds mainly originated from *Ranunculus ternatus* and *Picrasma quassioides* exhibited low binding free energies which below -9.0 kcal/mol. Compounds Japonicone G and Picrasidine T were obtained with favorable drug-likeness. Moreover, the complex of Japonicone G and Mpro had prominent stability.

**Conclusion:**

Natural compound Japonicone G is highly promising as a potent inhibitor against SARS-CoV-2 for further study.

## Graphical abstract

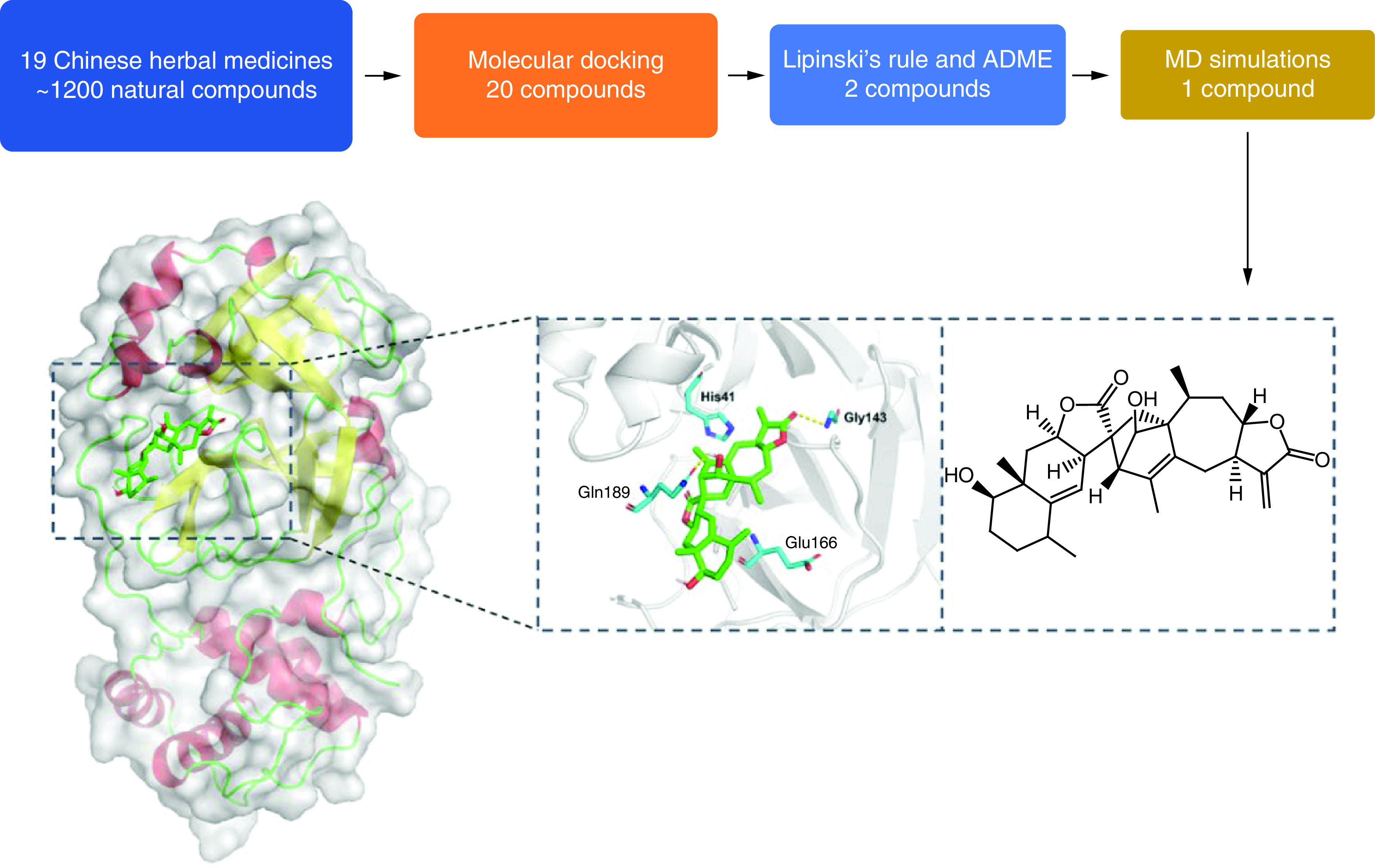

The novel coronavirus disease 2019 (COVID-19), caused by severe acute respiratory syndrome coronavirus 2 (SARS-CoV-2), has a serious impact on all walks of life and even human lives. It has been officially defined as a pandemic by the World Health Organization (WHO) on 11 March 2020 [1]. Typical symptoms of SARS-CoV-2 infection cover fever, dry cough, shortness of breath, weakness, etc. Severe cases can rapidly develop into acute respiratory distress syndrome and septic shock or heavier complications [2]. As of 15 July 2022, a total of 557,917,904 cases of COVID-19 has been confirmed worldwide, including 6,358,899 deaths (https://covid19.who.int/). Considering the severity of the pandemic, massive efforts have been devoted to study SARS-CoV-2. As a result, various preventive vaccines, special drugs and therapeutics have been developed [3]. However, new variants are continuously occurred as mutations in SARS-CoV-2 emerge frequently during viral replication [4]. Thus, effective inhibitors for SARS-CoV-2 are still highly desirable for providing more options and opportunities for the treatment of COVID-19.

Belonging to the coronavirus (CoV) family, SARS-CoV-2 is an enveloped, positive-sense, single-stranded RNA virus. The genome sequence of SARS-CoV-2 keeps 79 and 50% similarities with severe acute respiratory syndrome coronavirus (SARS-CoV) and Middle East respiratory syndrome coronavirus (MERS-CoV), respectively [5]. Polyproteins encoded by SARS-CoV-2 are processed into 16 nonstructural proteins (nsps) by the main protease (Mpro) and the papain-like protease (PLpro). These nsps contribute to the production of four main structural proteins that play an important role in the invasion and spread of SARS-CoV-2 in the human body [6,7]. In addition, the structures of Mpros were revealed to be relatively conserved among CoVs, especially in the substrate-binding pockets [8]. Correspondingly, Mpro sequences also show a low mutation frequency across known SARS-CoV-2 variants [9]. Hence, Mpro is an attractive target for the drug development against SARS-CoV-2.

As one of the most important components of traditional Chinese medicine (TCM), Chinese herbal medicine contains numerous natural compounds, and some of which have played key roles in the medicine industry [10]. Some of them already showed excellent efficacy against certain diseases. However, the efficacy mechanism of most Chinese herbal medicines is unelucidated. Up to now, a part of active ingredients from Chinese herbal medicines have been applied to the treatment of SARS-CoV-2 infections, which shows that it is promising to exploit novel inhibitors of SARS-CoV-2 from them [11,12].

Computational approaches are frequently used to estimate the potential inhibitors that could prevent the activity of a protein. So far many reported studies were developed by using *in silico* techniques to explore the resolutions of this pandemic caused by SARS-CoV-2 [13–18]. These computational methods, such as molecular docking, molecular dynamic simulations and molecular mechanics Poisson–Boltzmann surface area analysis, remarkably reduce the time and cost of drugs discovery and repurposing [19,20].

The study aims to determine natural compounds with inhibitory effects toward SARS-CoV-2 Mpro from Chinese herbal medicines through combined computational methods. Herein, to determine novel compounds with inhibitory effects toward SARS-CoV-2 Mpro, a host of natural compounds from Chinese herbal medicines listed in The National Collection of Chinese Herbal Medicine were collected in various ways and their binding affinities were studied. Molecular docking was used to rapidly assess the binding free energy of each compound with Mpro and identify potential inhibitors that performed well compared with previously reported inhibitors. Lipinski's rule and ADME (absorption, distribution, metabolism and excretion) were used to evaluate the drug-likeness and bioactivity of these compounds. Molecular dynamics simulation and molecular mechanics Poisson–Boltzmann surface area was carried out to further estimate the stability of the screened compounds in complex with Mpro. The results obtained could help advance the development of SARS-CoV-2 treatments.

## Materials & methods

### Preparation of natural compounds & protein

In this study, we collected ∼1200 natural compounds from 19 Chinese herbal medicines (Supplementary Table 1) via PubChem (https://pubchem.ncbi.nlm.nih.gov/), TCMSP (https://old.tcmsp-e.com/tcmsp.php) or manual drawn according to the results of our previous literature survey [21,22]. All these compounds were added hydrogens, generated 3D structures, converted into .pdb format and optimized in MMFF94s force field by OpenBabel (version 3.1.1) [23]. Then, we used AutoDockTools (ADT, version 1.5.6) to detect roots and choose torsions of these processed structures, saving them as .pdbqt format as well [24].

The 3D crystal structure of SARS-CoV-2 Mpro (PDB ID: 7VH8, 1.59 Å) was downloaded from RCSB Protein Data Bank (https://www.rcsb.org/) in .pdb format [9,25]. PyMol (version 2.5.0) was used to remove water molecules and the original inhibitor of the PDB structure [26]. Swiss-PdbViewer (version 4.1.0) was used to add missing residues in the protein [27]. ADT was used to add hydrogens and convert the structures into .pdbqt format.

### Molecular docking analyses

As a popular and free docking program, AutoDock Vina has a better success rate in detecting the highest-affinity ligand for a given target protein [28,29]. To rapidly and accurately evaluate the binding affinities and poses of numerous compounds with SARS-CoV-2 Mpro, the molecular docking by the AutoDock Vina (version 1.1.2) was employed with the exhaustiveness of 10. Semi-flexible doking was applied, that is, the Mpro was rigid and the natural compounds were flexible. The size of the docking grid box was 26 × 30 × 26 Å^3^, centered on (-19.952, 17.263, -23.28). The docking results for 3D and 2D were visualized by PyMol and LigPlot^+^ (version 2.2.5), respectively [30].

### Profiling of drug-likeness & ADME

The evaluation of drug-likeness is greatly important in the early stage of drug discovery. In this section, Lipinski's rule was adopted to estimate the drug-likeness properties of selected natural compounds [31]. The ADME properties determine the concentration, tissue distribution and metabolic pathway of a drug in the body, and have very important reference value for predicting the bioavailability and biological activity of a drug. Compounds that passed Lipinski's rule and ADME properties were predicted by specific parameters and indicators to further screen out compounds with desirable bioactivity. The SwissADME server (http://www.swissadme.ch/) was implemented for calculating the Lipinski's rule and the ADME properties as well [32].

### Molecular dynamics simulations

Molecular dynamics (MD) simulations for complexes of screened compounds with SARS-CoV-2 Mpro were carried out by Gromacs (version 2022) [33]. Charmm36-jul2021 force field and TIP3P water model were applied for each MD simulation [34]. The SwissParam server (https://www.swissparam.ch/) was used to generate the ligand topology [35]. The system was run in a dodecahedron water box and the protein was in the center of the box with a minimum distance no closer than 1 nm from the boundary during the simulation. 4 Na^+^ were added to neutralize the system charge. To make the simulation run successfully, the system was energy minimized by the steepest descent algorithm followed the conjugate gradient algorithm and 1339 steps were run to minimize the system. NVT and NPT equilibration were performed sequentially for 100 ps each and the temperature was set to 300 K at a pressure of 1 bar. Finally, MD simulations were carried out for 100 ns with a sampling interval of 10 ps. The binding free energy of the dynamically stable complex obtained from MD simulation was calculated through molecular mechanics Poisson-Boltzmann surface area by using the gmx_MMPBSA tool (version 1.5.7) [36].

## Results

### Potential inhibitors obtained by molecular docking

**Figure 1.**
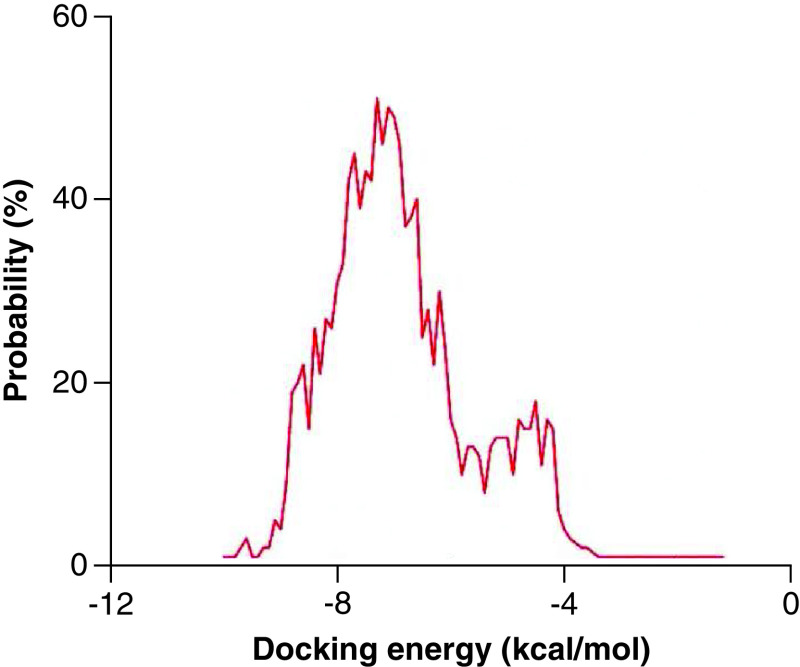
Distribution of docking energy between ∼1200 natural compounds and SARS-CoV-2 Mpro.

To ensure the reliability of docking results, two previously reported Mpro inhibitors [37], PF-00835231 and PF-07321332 were docked with SARS-CoV-2 Mpro under the set parameters, respectively [38]. The binding free energy of PF-00835231 (-8.3 kcal/mol) and PF-07321332 (-9.6 kcal/mol) both showed good performance. To strike a balance between the quality and quantity of the molecular docking screening results, -9.0 kcal/mol, the mean value of the binding free energies of PF-00835231 and PF-07321332, was chosen as a screening line. The top 20 compounds with binding free energies below -9.0 kcal/mol were selected and listed in Table 1. It can be observed that the top 20 compounds mainly from *Ranunculus ternatus* and *Picrasma quassioides*, specifically, eight of them are from the *Ranunculus ternatus*, six are from the *Picrasma quassioides*. The other six molecules are Japonicone G, Aristolochine, Protostemonine, Stemocochinin, 4′,5-Dihydroxyflavanone and Luteoloside, where Japonicone G is from *Inulae Herba*, Aristolochine is from *Aristolochia debilis*, Protostemonine and Stemocochinin are from *Stemona japonica*, 4′,5-Dihydroxyflavanone is from *Hydrangea febrifuga*, and Luteoloside is from *Gleditsia sinensis*. Structural information for all these 20 natural compounds was provided in Supplementary Table 3. The total docking energy ranges from -1.2 to -10.0 kcal/mol with an average value of -7.1 kcal/mol (Figure 1).

**Table 1.** Docking scores of top 20 compounds with SARS-CoV-2 Mpro and their Chinese herbal medicine sources.

S. no.	Compound name	Docking score (kcal/mol)	Latin name
1	Japonicone G	-10.0	*Inulae Herba*
2	Bilobetin	-9.9	*Ranunculus ternatus*
3	Ternatoside D	-9.7	*Ranunculus ternatus*
4	Aristolochine	-9.6	*Aristolochia debilis*
5	Kumujansine	-9.6	*Picrasma quassioides*
6	Robustaflavone-4′-methyl ether	-9.6	*Ranunculus ternatus*
7	Amentoflavone	-9.5	*Ranunculus ternatus*
8	Podocarpusflavone A	-9.4	*Ranunculus ternatus*
9	Quassidine G	-9.3	*Picrasma quassioides*
10	Protostemonine	-9.3	*Stemona japonica*
11	Picrasidine N	-9.2	*Picrasma quassioides*
12	Stemocochinin	-9.2	*Stemona japonica*
13	4’,5-Dihydroxyflavanone	-9.1	*Hydrangea febrifuga*
14	Kayaflavone	-9.1	*Ranunculus ternatus*
15	Isoginkgetin	-9.1	*Ranunculus ternatus*
16	Picrasidine M	-9.1	*Picrasma quassioides*
17	5,6,9,11-tetradehydrotoonaciliatin K	-9.1	*Picrasma quassioides*
18	Picrasidine T	-9.0	*Picrasma quassioides*
19	Luteoloside	-9.0	*Gleditsia sinensis*
20	Ternatoside C	-9.0	*Ranunculus ternatus*

**Figure 2.**
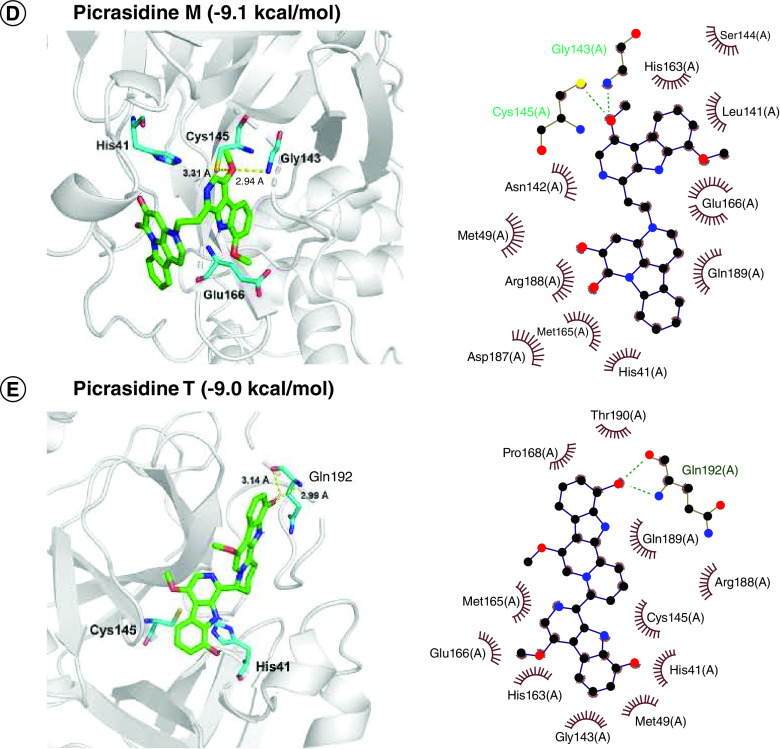

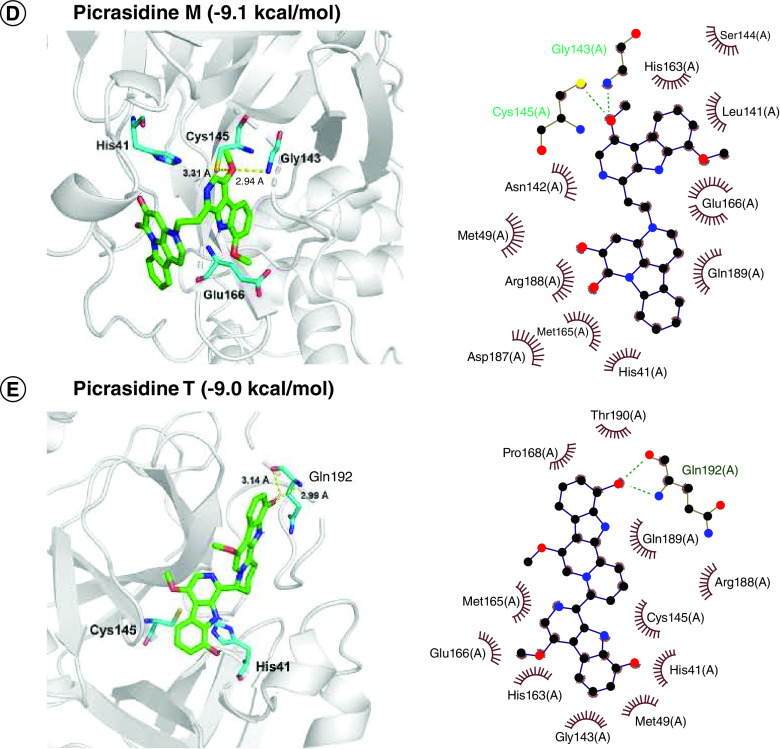
Docked comformations of six selected compounds with SARS-CoV-2 Mpro. Docked comformations of **(A)** Japonicone G, **(B)** Kumujansine, **(C)** Quassidine G, **(D)** Picrasidine M and **(E)** Picrasidine T within the active site of SARS-CoV-2 Mpro along with their corresponding 2D interaction plots.

Molecular docking interactions of six natural compounds (Japonicone G, Kumujansine, Quassidine G, Picrasidine M and Picrasidine T) were shown in Figure 2. It can be observed from Figure 2A that Japonicone G (CAS Number: 1228171-27-4) with the lowest interaction energy (-10.0 kcal/mol) formed hydrogen bonds with the residues Gly143 (2.90 Å) and Gln189 (2.91 Å) in the binding site that contains the catalytic dyad formed by His41 and Cys145. Hydrophobic interactions were found between the amino acid residues and Japonicone G at His41, Met49, Asn142, Met165, Glu166, Pro168, Asp187, Arg188 and Thr190 in the 2D interaction plot of Figure 2A.

The interaction energies of compounds Kumujansine (-9.3 kacl/mol), Quassidine G (-9.1 kcal/mol), Picrasidine M (-9.0 kcal/mol) and Picrasidine T (-9.0 kal/mol) were higher than that of Japonicone G. In Figure 2B, Kumujansine had hydrophobic interactions with many surrounding amino acid residues like Thr25, His41, Met49, Leu141, Asn142, Gly143, Ser144, Cys145, His163, Met165, Glu166, etc. Quassidine G interacted with residue Arg188 via a hydrogen bond at a distance of 3.07 Å and had hydrophobic interactions with residues Phe140, Leu141, Asn142, Ser144, Cys145, His 163, Met165, Glu166, Pro168, Asp187, Gln189, Thr190 and Gln192 (Figure 2C). Picrasidine M could interacted with the residue Gly143 (2.94 Å) or Cys145 (3.31 Å) through hydrogen bonds and hydrophobic interactions occurred at His41, Met49, Leu141, Asn142, Ser144, His163, Met165, Glu166, Asp187, Arg188 and Gln189 (Figure 2D).

In Figure 2E, Picrasidine T, which had a binding free energy of -9.0 kcal/mol, interacted with the residue Gln192 via hydrogen bonds. Hydrophobic interactions were also found between the amino acid residues and Picrasidine T at His41, Met49, Gly143, Cys145, His163, Met165, Glu166, Pro168, Arg188, Gln189 and Thr190. Although these top 20 natural compounds showed lower binding free energy and higher binding affinity when docked with SARS-CoV-2 Mpro, their physicochemical properties and binding stability of complexes still need further testing.

### Lipinski's rule of selected compounds

Further these 20 natural compounds selected with molecular docking results were evaluated by Lipinski's rule, involving molecular weight, rotatable bonds, hydrogen bonds acceptors, hydrogen bonds donors and MLogP. It can be considered to have better drug-likeness properties when the Lipinski's violations of a compound are less than one. As shown in Supplementary Table 4, 16 of 20 compounds obeyed the Lipinski's rule, except compounds Ternatoside D and 4′,5-Dihydroxyflavanone with three Lipinski violations, Amentoflavone and Luteoloside with two Lipinski's violations. This suggested that most of the natural compounds in Chinese herbal medicines have high medicinal potential.

### ADME properties of selected compounds

To further evaluate the bioavailability of selected compounds, ADME properties of 16 screened natural compounds and PF-07321332 were carried out by swissADME as shown in Supplementary Table 5. The iLogP value reflects the lipophilicity of the compound, which can affect the absorption of the drug [39]. The lower the iLogP value means the better drug absorption effect. The Log S (ESOL, estimated solubility) shows the water solubility of a compound [40]. When the value of Log S is less than -10, it means that the compound is insoluble, -10 to -6 means poorly soluble, -6 to -4 means moderately soluble and -4 to -2 means soluble. In Supplementary Table 5, compounds Japonicone G, Kumujansine, Quassidine G, Protostemonine, Picrasidine N, Stemocochinin, Picrasidine M and Picrasidine T were all moderately soluable, except that Ternatoside C was soluable only. Gastrointestinal (GI) absorption was predicted by the white of the BOILED-Egg (brain or intestinal estimated permeation predictive model) and blood-brain barrier (BBB) permeation by the yolk of that [41]. The effect of GI absorption plays a critical role in the treatment of diseases, especially severe cases that are difficult in absorbing drugs [42]. A higher GI value usually equates to a better therapeutic effect. The BBB protects the brain from foreign substances in the blood and maintains the stability of the brain environment [43]. Among compounds with moderate or higher solubility, Japonicone G, Kumujansine, Quassidine G, Picrasidine N, Picrasidine M and Picrasidine T had both high GI absorption and no BBB permeant.

CYP1A2, CYP2C19, CYP2C9, CYP2D6 and CYP3A4 are members of cytochrome P450 enzymes, which are responsible for the metabolism of the majority of drugs and play an important role in the biotransformation of drugs, especially the 1, 2 and 3 CYP-families [44]. In Supplementary Table 5, Japonicone G was not an inhibitor of 5 CYPs assessed. Kumujansine and Quassidine G were inhibitors of 4 of 5 CYPs. Picrasidine N and Picrasidine M were inhibitors of 3 CYPs, and Picrasidine T was 2.

As a positive control, PF-07321332 had a relatively low iLOGP value (3.01) and was soluble with a -3.58 Log S value. Meanwhile, it possessed high GI absorption, no BBB permeant and just an inhibitor of CYP3A4. From Supplementary Table 5, it can be obtained that each selected compounds had a bioavailability score of 0.55, and with the PF-07321332 as the control, Japonicone G possessed the favorable pharmacokinetic profiles, followed by Picrasidine T.

### MD simulations of two screened compounds

**Figure 3.**
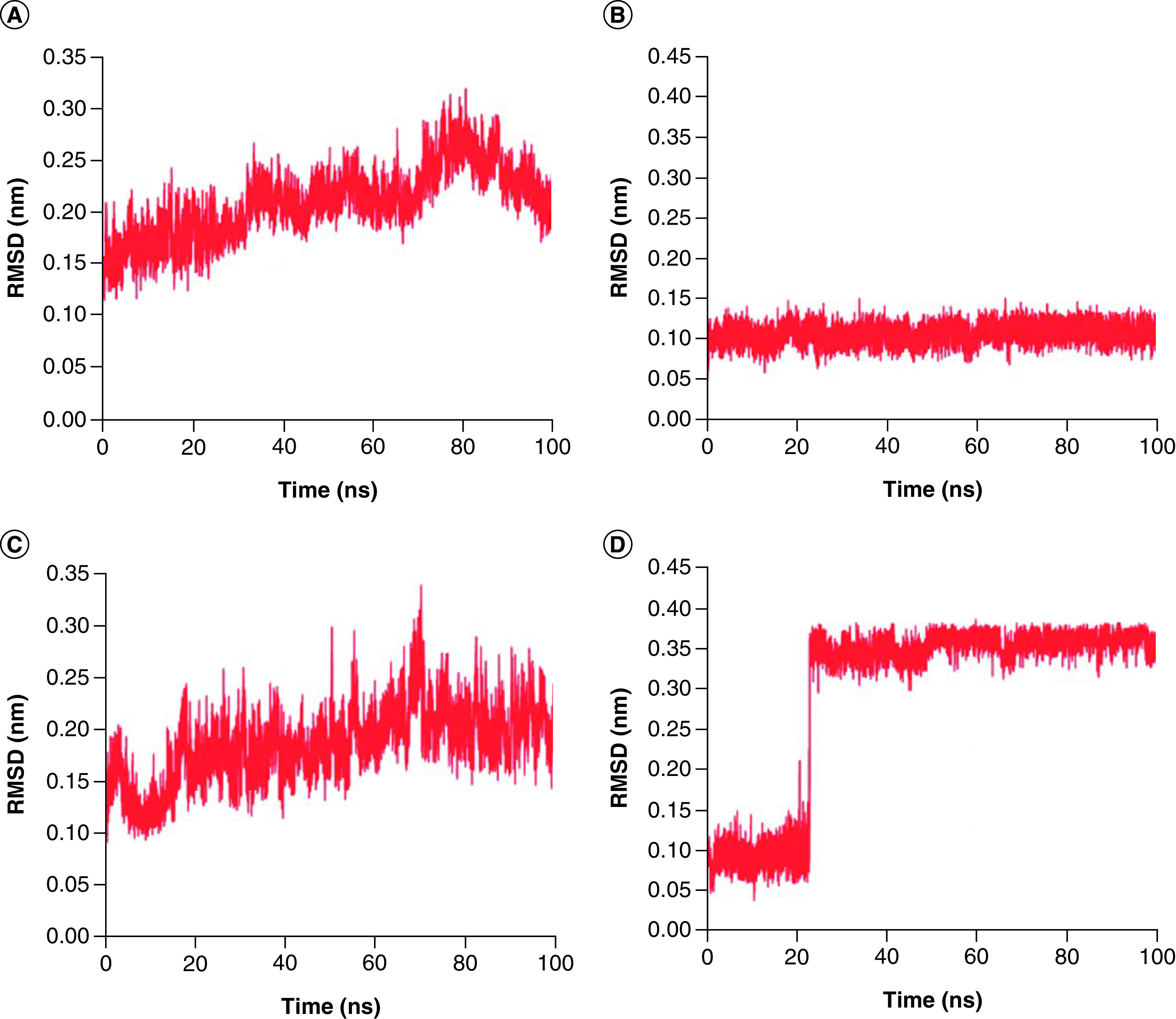
RMSD plot of two complexes. RMSD plot of complex Japonicone G and Picrasidine T with SARS-CoV-2 Mpro during 100 ns MD simulation, respectively: **(A)** Mpro in complex with Japonicone G; **(B)** Japonicone G in complex; **(C)** Mpro in complex with Picrasidine T; **(D)** Picrasidine T in complex.

To determine the more stable complex between two complexes: Japonicone G with SARS-CoV-2 Mpro and Picrasidine T with SARS-CoV-2 Mpro, two separate MD simulations for each complex were carried out (Supplementary Figure 1). Root mean square deviation (RMSD), root mean square fluctuation (RMSF), radius of gyration (Rg) and hydrogen bond (Hbond) were analyzed to further determine the dynamics and stability of each docking complex. The RMSD value reflects the deviation from the initial structure and can be used as a measure of the overall stability for the complex [45]. From Figure 3A & C, it can be observed that Mpro in both complexes remained a relatively stable state after the initial fluctuation, with RMSD average values of 0.21 nm and 0.19 nm, respectively, indicating that neither compound significantly changed the stability of the Mpro structure. The RMSD of Japonicone G in its complex had almost always maintained steady with an average value of 0.11 nm (Figure 3B). For the case of Picrasidine T, an increase appeared at ∼23 ns by leaps and bounds, and then the average value of its RMSD remained at 0.36 nm, which was almost three-times that of Japonicone G.

**Figure 4.**
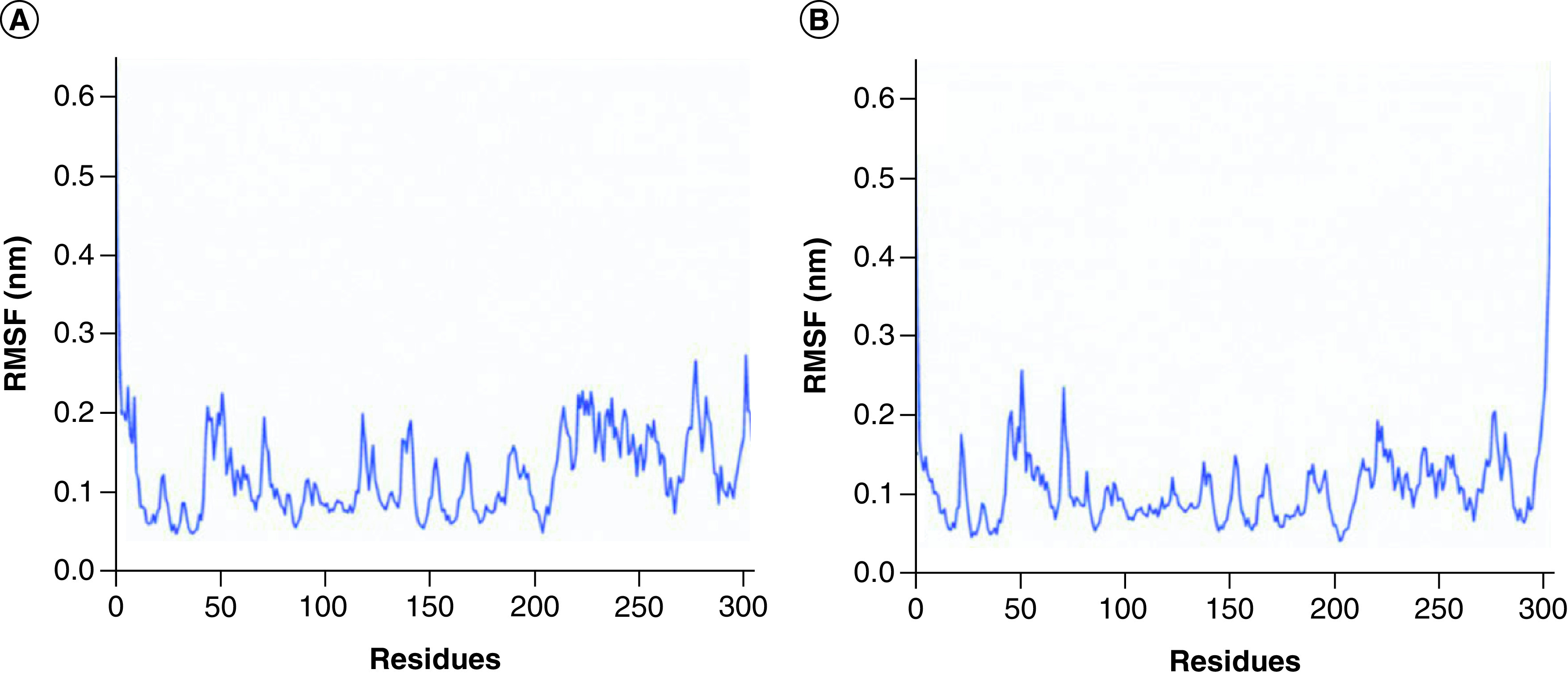
RMSF plot of SARS-CoV-2 Mpro in two complexes. RMSF plot of SARS-CoV-2 Mpro (C-alpha atom) in two complexes during 100 ns MD simulations, respectively: **(A)** Mpro in complex with Japonicone G; **(B)** Mpro in complex with Picrasidine T.

The RMSF values of Mpro based on C-alpha atom were calculated to investigate the flexibility of residues [46]. In Figure 4A, Mpro in complex with Japonicone G fluctuated more clearly around residues Pro52, Asn72, Asn119, Asn142 and domain III (residues 201–303). In Figure 4B, Mpro in complex with Picrasidine T fluctuated more clearly around residues Pro52, Asn72 and domain III. The average RMSF value of the former was 0.12 nm (Figure 4A) and that of the latter was 0.11 nm (Figure 4B), which was consistent with that RMSD value of the former was slightly higher than the latter.

**Figure 5.**
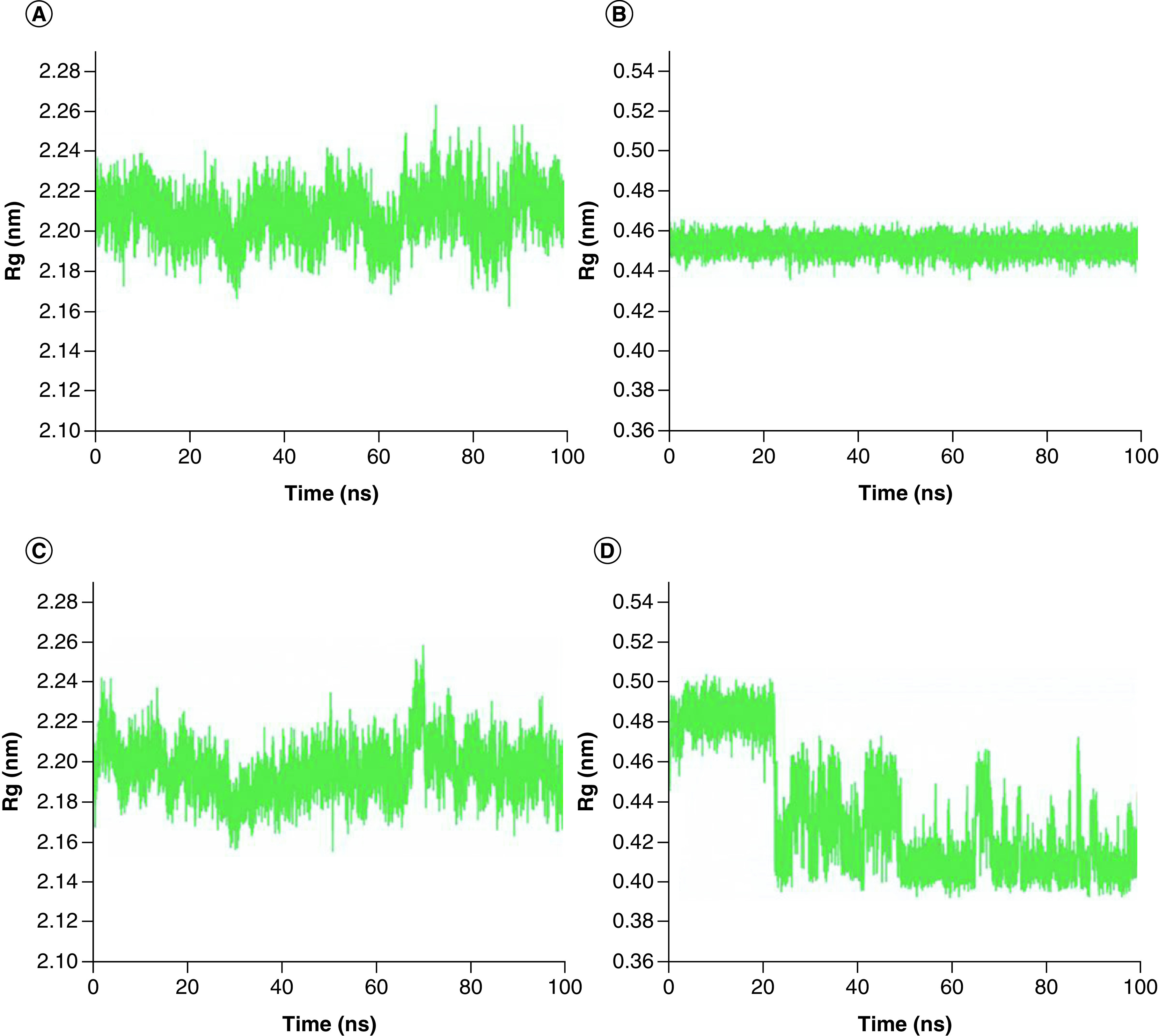
Rg plot of two complexes. Rg plot of complex Japonicone G and Picrasidine T with SARS-CoV-2 Mpro during 100 ns MD simulation, respectively: **(A)** Mpro in complex with Japonicone G; **(B)** Japonicone G in complex; **(C)** Mpro in complex with Picrasidine T; **(D)** Picrasidine T in complex.

To examine the compactness degree of the structure, the Rg values were calculated [47]. From Figure 5A & C, it can be noticed that Mpro in both compelxes keep stable overall, with Rg values of 2.21 nm and 2.20 nm, respectively. The average Rg value of Japonicone G in its complex was 0.45 nm and its standard deviation was 0.004 nm (Figure 5B), which implied that the structure of Japonicone G held extremely stable throughout the MD simulation. However, for the Rg values of Picrasidine T (Figure 5D), an obvious decrease appeared at 23 ns, demonstrating that the structure of Picrasidine T altered and became more compact at this time, which corresponds to the sharp increase of its RMSD value in Figure 3D.

**Figure 6.**
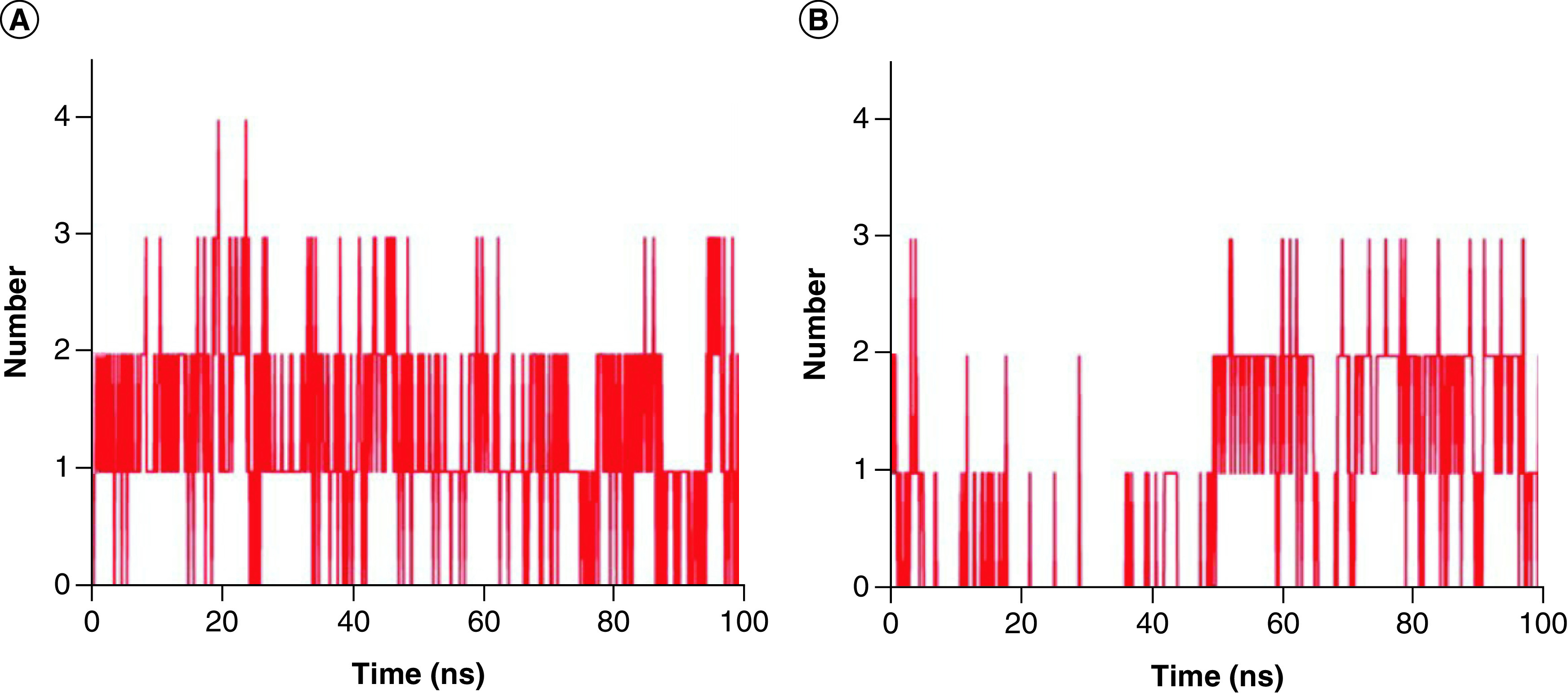
Hydrogen bond number plot of two complexes. Hydrogen bond number plot of complex **(A)** Japonicone G and **(B)** Picrasidine T with SARS-CoV-2 Mpro during 100 ns MD simulation, respectively.

During the MD simulation, the ligand forms a certain number of hydrogen bonds with the protein, which can also reflect the stability and strength of the binding complex. In Figure 6A, Japonicone G formed at least 0 and at most 4 hydrogen bonds with SARS-CoV-2 Mpro during the 100 ns MD simulation. In the case of Picrasidine T a minimum of 0 hydrogen bond and a maximum of 3 hydrogen bonds were found (Figure 6B). Overall, it can be observed from Figure 6 that during the simulation, Japonicone G formed more hydrogen bonds that of Picrasidine T, which was also consistent with the results of molecular docking.

### MMPBSA analysis

The MMPBSA calculation was performed to further strengthen the findings [19]. The binding free energy of Japonicone G in complex with SARS-CoV-2 Mpro based on MD simulations was calculated. From Table 2, it can be found that the binding energy of the complex was -22.13 kcal/mol, which shows that the complex of SARS-CoV-2 with Japonicone G was very stable. The van der Waals energy is the major advantageous contributors toward binding energy, followed by electrostatic energy, while the polar solvation energy contributed unfavorably toward the binding of Japonicone G with Mpro. To further clarify the pivotal residues involved in the complexation process, the binding free energy was decomposed into the energy contribution of each residue [48].

**Table 2.** Contribution of each energy element (in kcal/mol) for the interaction of SARS-CoV-2 Mpro with Japonicone G.

van der Waal energy	Electrostatic energy	Polar solvation energy	SASA energy	Binding energy
-39.12	-11.11	31.94	-3.85	-22.13

**Figure 7.**
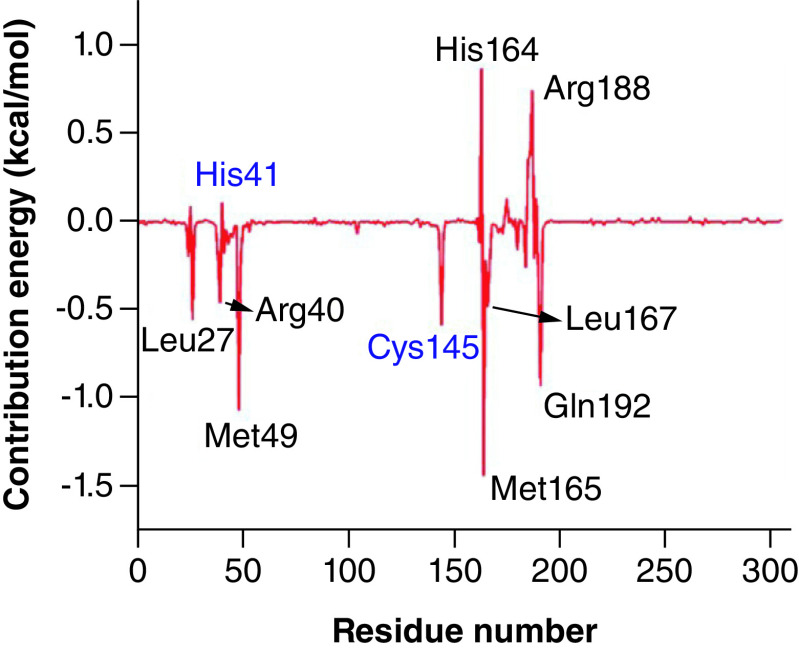
Contribution energy per amino acid residue of SARS-CoV-2 Mpro upon interaction with Japonicone G.

In Figure 7, it could be observed that the residues such as Leu27, Arg40, His41, Met49, Cys145, His164, Met165, Leu167, Arg188 and Gln192 actively participated in the complexation process. Most of these residues contribute negative binding energy, which played a crucial role in stabilizing the complex, while few residues had a positive binding energy contribution.

## Discussion

In our present study, we performed multiple computational methods to screen ∼1200 natural compounds from 19 Chinese herbal medicines and found that Japonicone G had the best binding affinity and stability in complex with SARS-CoV-2 Mpro.

To rapidly identify the most effective inhibitors of SARS-CoV-2 Mpro, we used molecular docking and obtained the top 20 natural compounds with favorable binding free energies compared with previously reported inhibitors, PF-00835231 and PF-07321332 [37]. It is worth mentioning that before the formal molecular docking of all the natural compounds, we tested our set docking parameters with these two previously reported inhibitors. According to a previous study, the binding free energy below -7.0 kcal/mol could be effective against SARS-CoV-2 Mpro [49]. The binding free energy of PF-00835231 (-8.3 kcal/mol) and PF-07321332 (-9.6 kcal/mol) both showed good performance, which were consistent with the experimental data that both inhibition constants of them were very low (Supplementary Table 2). Therefore, this method was reliable and provided a believable evaluation of all these ∼1200 natural compounds. The average value of total docking energy is -7.1 kcal/mol, which corroborates that Chinese herbal medicines are remarkably desirable for exploiting novel inhibitors of SARS-CoV-2 [11,50].

Considering drug-likeness and pharmacokinetic properties assessed by Lipinski's rule and ADME, the docked poses of six selected compounds were displayed in Figure 2. Among all docked natural compounds, Japonicone G possessed the lowest binding free energy (-10.0 kcal/mol) and generated hydrogen bonds with the residues Gly143 and Gln189, which were also reported before in the inhibitor N3 docking with SARS-CoV-2 Mpro [51]. No hydrogen bonds were found between Kumujansine and other residues, but more hydrophobic interactions were formed between Kumujansine and 17 residues. Hydrophobic interactions can make improvement on binding affinity and increase the bioactivity of the compound, which should be partly responsible for the binding free energy of -9.6 kcal/mol for Kumujansine [52]. Quassidine G formed a hydrogen bond with residue Arg188, which was demonstrated in a recent study [53]. For the hydrogen bonds that can be generated by Picrasidine M and two residues Gly143, Cys 145, respectively, the former residue was similar to that of Japonicone G, but at a slighter longer distance, and the residue Cys145 involved in the latter is part of the catalytic dyad [54]. Picrasidine T formed a hydrogen bond with residue Gln192, which was also detected in phytochemicals from *A. paniculate* in previous research of SARS-CoV-2 Mpro [55]. Furthermore, as shown in Figure 2, all six compounds had hydrophobic interactions with surrounding amino acid residues, especially Met165 and Glu166.

Lipinski's rule and ADME parameters were applied to evaluate the drug properties of the compounds screened by molecular docking. For the top 20 selected compounds, 16 satisfied Lipinski's rule, and then the ADME parameters further revealed whether their physicochemical properties meet the standards of drugs [56]. Compared with PF-07321332, the ADME parameters of the most selected compounds were not as favorable as it, except Japonicone G and Picrasidine T. Although the iLOGP value of Japonicone G (3.55) was mildly higher than that of PF-07321332 (3.01) and its ESOL Class was MS, it was not an inhibitor of 5 CYPs assessed, in the case that their GI absorption, BBB permeant, bioavailability score were all consistent, while PF-07321332 was a CYP3A4 inhibitor. In addition, Picrasidine T, with an iLOGP value of 2.96 and inhibitors of 2 CYPs, could be almost compared with the positive control.

To further evaluate the stability of the two docking complexes: Japonicone G with SARS-CoV-2 Mpro and Picrasidine T with SARS-CoV-2 Mpro, we carried out MD simulations and analyzed RMSD, RMSF, Rg and Hbond of each complex. The results showed that Japonicone G in complex with Mpro had higher stability during the 100 ns MD simulation. To farther strengthen the findings, we performed the MMPBSA analysis for the complex of Mpro with Japonicone G. The total binding energy of the complex was -22.13 kcal/mol, which showed that the binding affinity of Mpro and Japonicone G was very strong [36]. Except the polar solvation energy contributed adversely to the total binding energy, all the other energy elements made a beneficial influence and among which van der Waals energy was the major contributor. From the plot of contribution energy for per residue, the residues like Met165 (-1.44 kcal/mol), Met49 (-1.07 kcal/mol) and Gln192 (-0.93 kcal/mol) had the lowest binding energy among all the residues.

In this work, natural compounds were screened out by combined computational methods so that *in vivo* and *in vitro* experiments are necessary in the future. Our results indicated that Japonicone G can be used as a potent inhibitor against SARS-CoV-2 for further study and its structure may provide new inspirations for anti-SARS-CoV-2 drug design.

## Conclusion

Among ∼1200 natural compounds from 19 Chinese herbal medicines in the molecular docking study, the top 20 compounds showed appropriate binding free energies with the active site of Mpro, which were mainly from the *Ranunculus ternatus* and *Picrasma quassioides*. Lipinski's rule and ADME analyses indicated that Japonicone G had the best binding affinity and drug-likeness, followed by Picrasidine T. MD simulations further showed that the binding complex of Japonicone G with SARS-CoV-2 Mpro had higher stability than that of Picrasidine T. Besides, MMPBSA calculation indicated the strong affinity of the binding of Japonicone G and SARS-CoV-2 and indicated that the residue Met165, Met49 and Gln192 played a major role in stabilizing the complex formed. Therefore, Japonicone G can be considered as a competitive candidate for the drug development against SARS-CoV-2. Given that this work was carried out by computational methods, further *in vivo* and *in vitro* experiments are necessitated to ensure the availability of Japonicone G for practical therapeutics.

**Summary points**

- Since the outbreak of Coronavirus disease 2019 (COVID-19) around the world, there is no acknowledged definite and effective treatment for COVID-19 although some drugs are under investigation.
- Due to structural conservation and low mutation frequency, Mpro is a critical drug target for treating SARS-CoV-2 infections.
- Chinese herbal medicines contain numerous and unexploited natural compounds, which are expected to have effective inhibitors against SARS-CoV-2.
- Computational methods, including molecular docking, drug-likeness assessment and molecular dynamics simulation, were combined to obtain potent inhibitors against SARS-CoV-2 Mpro.
- 20 natural compounds show favorable binding affinities to Mpro.
- Japonicone G had the best binding affinity and drug-likeness, followed by Picrasidine T.
- Japonicone G binds into active site of Mpro by hydrogen bonding and hydrophobic forces and the complex had prominent stability.
- Two kinds of Chinese herbal medicines, the *Ranunculus ternatus* and *Picrasma quassioides*, were found to contain many natural compounds with inhibitory effects against SARS-CoV-2 Mpro.
